# Development of Garments with Elastic Straps and Pressure Applicator (GESPA) and “GVcorrect” App to Follow the Changes in Lower-Extremity Alignment (*Genu Valgum*)—A Pilot Study

**DOI:** 10.3390/pediatric13030057

**Published:** 2021-08-10

**Authors:** Anna-Liisa Tamm, Ivi Vaher, Reet Linkberg, Teet Tilk, Jana Kritt, Age Alviste, Maarit Sild, Arved Vain

**Affiliations:** 1 Physiotherapy and Environmental Health Department, Tartu Health Care College, Nooruse 5, 50411 Tartu, Estonia; ivivaher@nooruse.ee (I.V.); reetlinkberg@nooruse.ee (R.L.); age.alviste@gmail.com (A.A.); maaritsild@gmail.com (M.S.); 2 Institute of Technology, University of Tartu, Nooruse 1, 50411 Tartu, Estonia; teet.tilk@ut.ee; 3 Tallinn Children’s Hospital Foundation, Tervise 28, 13419 Tallinn, Estonia; jana.kritt@lastehaigla.ee; 4 Institute of Physics, University of Tartu, W. Ostwaldi 1, 50411 Tartu, Estonia; arved.vain@ut.ee

**Keywords:** genu valgum, children, mechanotherapy, tibiofemoral angle, intermalleolar distance, GESPA, GVcorrect app

## Abstract

Background: There are non-invasive methods of correcting *genu valgum* (GV), but to date, there is no method to evaluate mechanotherapeutic intervention that does not restrict child’s natural movements while the process is on-going so that timely decisions could be made on effectiveness of intervention. The aim of study was to develop and assess the comfortability of garments with elastic straps and pressure applicator (GESPA) and the reliability and user-friendliness of “GVcorrect” app, which aims to catch the elastic straps’ pressure level (mN). Methods: 6 children (5–7 y) with intermalleolar distance ≥5 cm wore GESPA daily for 3 months. Anthropometrical and goniometrical measurements were done according to standard technique; tone and biomechanical parameters of skeletal muscles determined with MyotonPRO; feedback about GESPA and “GVcorrect” collected via questionnaire. Results: Based on feedback from children and parents, new, more comfortable and user-friendly GESPA were designed; several updates were made to “GVcorrect” app; new goals were set for the next phase of the study. Conclusions: GESPA and the “GVcorrect” app serve their purpose, but there are still a number of important limitations that need to be removed before the product can be marketed. The study continues with product development until a medical device certificate is obtained.

## 1. Introduction

In 2- to 6-year-old children, *genu valgum* (GV) is normal within certain limits of tibiofemoral angle, therefore, being characterized as a physiological condition [1]. Development related changes usually correct themselves spontaneously [2]; so, by 7 to 11 years, knees return to a neutral position [3]. The prevalence of GV in growing children is approximately 7% [4] and it is mostly expressed as intermalleolar distance (IMD) [5] more than 5 cm [6,7] and tibiofemoral angle (TFA) < 162 degrees over the age of 7 [8]. From first grade children (6–8 y; *n* = 4427) in Tallinn (capital of Estonia), 1.76% had IMD ≥ 5 cm and 1.35% 4–4.9 cm [9].

GV prevalence is higher among overweight and obese children and approximately 70% of children with GV are obese [10]. As part of the European Childhood Obesity Surveillance (2015/2016), more than three-quarters of Estonian 1st grade students (mostly 7-year-old children) were measured and weighed and it turned out that every fourth girl and third boy were overweight or obese [11]. According to the International Survey on School Health Behavior, the share of overweight (incl. obese) 11-, 13- and 15-year-old students in Estonia has increased almost three times on average from 2001 to 2017 [12]. So, we could assume that the prevalence of GV is rising in Estonia. Permanent valgus deformities lead to consequences in daily activities as walking, sitting, raising and going up and down stairs [13] and cause arthrosis [14].

In cases of mild persistent GV can be successfully treated conservatively [2]. If the possibilities of conservative therapy have been exhausted, surgical leg straightening is the therapeutic standard [15]. The benefits (incl gentle and simple procedure; good or excellent outcomes) of gradual correction of fixed knee flexion deformity by guided growth using the flexible construct of the eight plate has several times documented [15,16,17]. There are also some complications associated with guided growth techniques: rebound phenomenon, inadequate reduction, implant breakage and implant migration [18].

There are also non-invasive methods of correcting GV (incl methods the principle of kinesio-tape; TheraTogs Ultra), but until now, there is no method to evaluate mechanotherapeutic intervention that does not restrict the child’s natural movements while the process is on-going so that timely decisions could be made on the effectiveness of the intervention. Scientists in Tartu Health Care College in collaboration with University of Tartu and The Health Clinic have been studied garments with elastic straps and pressure applicator (GESPA) [19,20] in children with *genu valgum* since 2014. The aim of this study-period (2020) was to develop and assess the comfortability of GESPA and the reliability and user-friendliness of “GVcorrect” app, which aims to catch the GESPA` elastic straps pressure level (mN).

## 2. Materials and Methods

The study is carried out as an applied research project in collaboration with Tartu Health Care College, University of Tartu and The Health Clinic [21]. There are 9 levels in the development of technology. This article provides an overview of the transition from technology readiness level 5 to level 6, which is responsible for verifying the functional readiness of the method and equipment. A relatively accurate prototype must be completed compared to the expected end product. Level 5 technology is thoroughly tested in the laboratory and in an appropriate simulated environment. The study was approved by Research Ethics Committee of National Institute for Health Development with identifier number 63 (2019). Informed consent was obtained from all subjects and their parents involved in the study.

### 2.1. Participants

Children aged 5–7 years with intermalleolar distance ≥5 cm were included (*n* = 6) whose lower limb alignment has not yet been fixed. The condition for inclusion was that the subjects were not involved in physical therapy and did not use orthoses during the study. Children with any diagnosed orthopedic pathology were excluded from the sample.

### 2.2. GESPA

**Figure 1:**
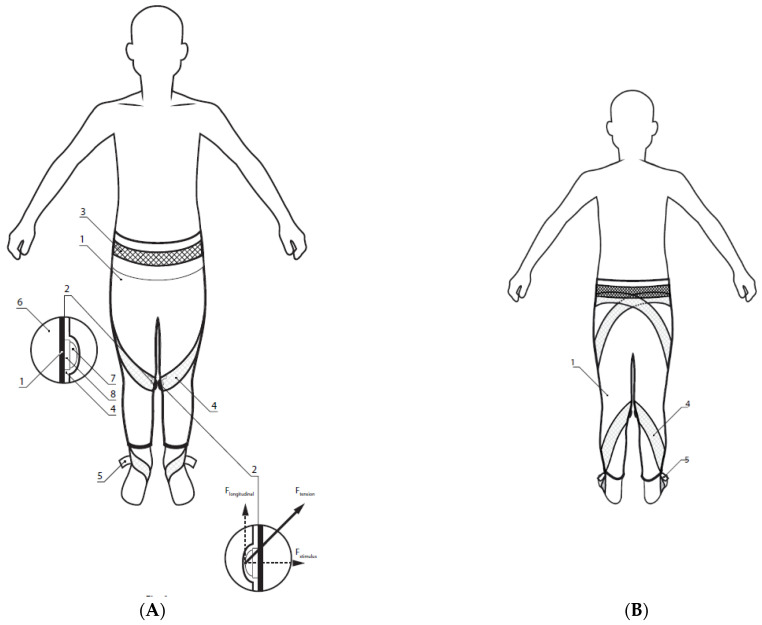
(**A**)—garments with elastic straps and pressure applicator (GESPA)—frontal-view: F_tension_—tension of elastic straps, F_stimulus_ (pressure)—force acting on the knee joint and F_longitudinal_—force acting on the sole of the foot; 1—garments; 2—pressure sensor and sensor’s pocket; 3—waistband of garments; 4—elastic straps; 5—adjuster for adjustment of stress in the elastic straps; 6—medial surface of knee joint; 7—hemisphere in contact with elastic straps; 8—pressure sensor. (**B**)—GESPA back-view: 1—garments; 4—elastic straps; 5—adjuster for adjustment of stress in the elastic straps.

**Figure 2:**
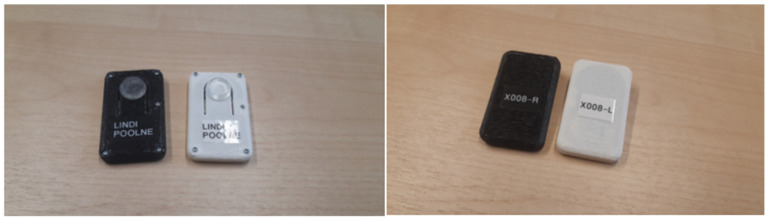
Frontal- and back-view of force pressure sensor.

Subjects of the study wore GESPA (manufacturer Ermiine LLC; Figure 1), which were sewn especially for correction each participant for three months at least six hours per day. The elastic straps are attached to the leg of the leggings and run helically in the closed channels of the leggings mounted on the left and right sides of the leg, crossing the pressure sensor (Figure 2) above the medial side of knee joint. The pressure sensor is located in the pocket of the sensor holder surrounding the knee joint. The pressure was computed between 500–1000 mN and it was adjustable with elastic straps running from under mid-foot (Figure 1(5)). GESPA does not restrict a child from performing all daily movements [20].

### 2.3. “GVcorrect”

“GVcorrect” is an application running on Android phones, which aims to catch the GESPA elastic band pressure level (mN). The app was developed in collaboration with software and product development partner Mooncascade [22]. The application works only with special treatments, which are used to correct the alignment of the user’s knee joint. “GVcorrect” allows the user to create an account, set monitoring criteria, connect to sensors and see the pressure on the sensors in real time. The application indicates a connection loss, a low sensor battery, or a pressure drop across the limits. The application works on Android phones from Android version 5.1 “Lollipop”, which supports at least “bluetooth” 4.2.

### 2.4. Data Collection

At the first measurement (before the intervention, **assessment I, AI**) and after (right after GESPA wearing period—**assessment II, AII**) the practical part anthropometric, goniometric parameters and myometric parameters: tone (frequency), biomechanical properties (stiffness, logarithmic decrement) of *m. sartorius*, *m. tensor fasciae latae*, *m. semitendinosus*, *m. tibialis anterior*, *m. gastrocnemius caput mediale*) were measured. Three months after wearing period the final measurement was done to evaluate long term effect of GESPA—**assessment III (AIII)**.

All anthropometrical (height, body mass, IMD) [23] and goniometrical (TFA) [24] measurements were done according to the standard technique in standing position. The child was barefoot, in as little clothing as possible. Body height was measured using a Harpender metal anthropometer to the nearest 0.1 cm; body mass with a medical electronic scale (A&D Instruments, Ltd., Abingdon, UK) to the nearest 0.1 kg; body mass index (BMI) was calculated (BMI = kg/m^2^); BMI was evaluated according to National Centre for Health Statistics [25]. To determine the tone, biomechanical parameters of skeletal muscles myometric method in multiscan mode was used. MyotonPRO (Myoton AS, Estonia) allow to assess the condition of the surface skeletal muscles safely, non-invasively, cost-effectively and in real time [26].

### 2.5. Feedback to the GESPA and “GVcorrect”

In AII and the follow-up period (AIII), parents in collaboration with children were asked to complete a questionnaire that reflected comfortability of GESPA and reliability and user-friendliness of “GVcorrect” app. The questionnaire consisted of 5 “yes or now” questions. They were asked to explain the answers during the short interview.

The researchers evaluated the user-friendliness of monitoring the data in the cloud server. Investigators monitored the appearance of data on the cloud server on a weekly basis and recorded sensor readings. If the data did not appear on the cloud server, the parents were contacted to find out if GESPA had been used. Each non-transfer was recorded.

### 2.6. Statistical Analysis

For estimating the skeletal muscle tone (oscillation frequency—Hz) and biomechanical properties (logarithmic decrement and dynamic stiffness N/m) individual analysis was carried out. MultiScan pattern of 20 measurements was used and the mean was considered [26]. The *p* value of < 0.05 was considered significant.

## 3. Results

### 3.1. Characteristics of Subjects

**Figure 3:**
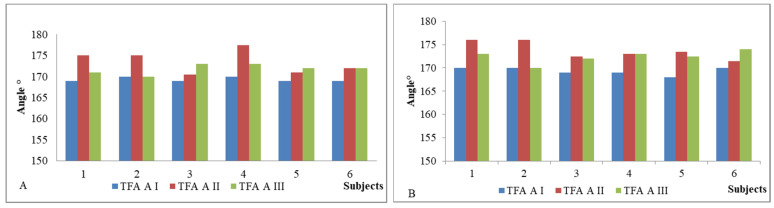
TFA values (left (**A**) and right (**B**) leg) in standing position in first (I), second (II) and third (III) assessment.

**Figure 4:**
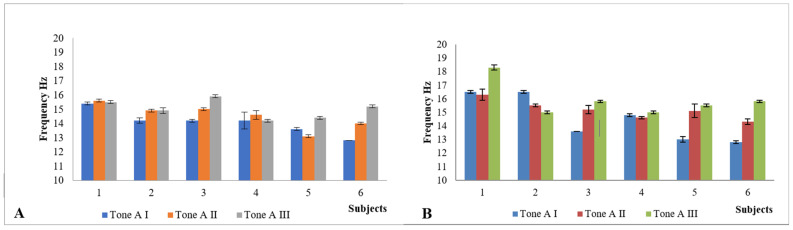
The tone of *m*. *sartorius* (left (**A**) and right (**B**) leg) in first (I), second (II) and third (III) assessment.

**Figure 5:**
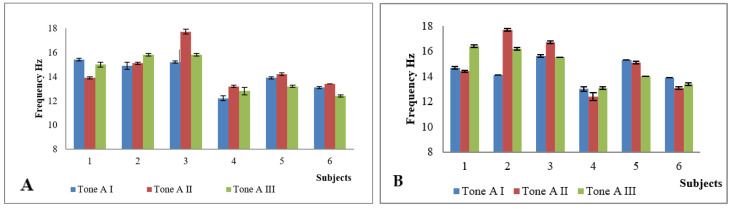
The tone of *m. tensor fasciae latae* (left (**A**) and right (**B**) leg) in first (I), second (II) and third (III) assessment.

Main characteristics of subjects (*n* = 6) of the study are presented in Table 1. Age, height, body mass and BMI were determined in AI. Only one participant (subject I) was male. According to National Centre for Health Statistics, subject I was obese, subjects IV and VI overweight and other three participants in normal weight. As it was important for us to get as much feedback as possible on the device and GESPA (including why the child did not want to wear GESPA), a child who only carried GESPA for five days was not excluded from the study (subject II). IMD values after the GESPA wearing period (AII) mostly decreased which shows the improvement of the alignment of the lower limb. TFA values in standing position in AI, AII and AIII are presented in Figure 3; the tone of *m*. *sartorius* in Figure 4; and the tone of *m. tensor fasciae latae* in Figure 5. In five subjects, the angle of TFA increased in both the left and right legs, in one subject there was no change (subject I). The angle value above baseline remained three months after the end of the study in three subjects and fell to baseline in one subject. There were only statistically significant changes in muscle parameters in terms of tone, with significant changes in all subjects (by the end of the GESPA wearing period and the end of the GESPA wearing free period). The changes in muscle tone studied during both the GESPA wearing period and the non-wearing period were statistically significant (*p* < 0.05).

**Figure 6:**
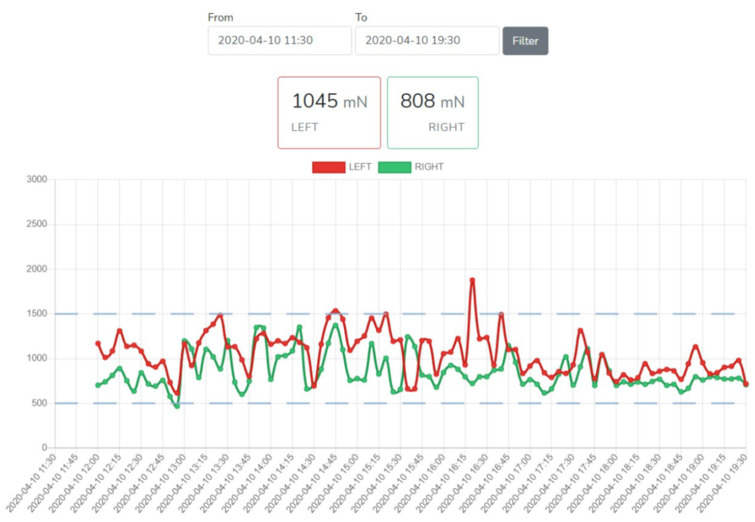
Cloud server view on computer screen (subject IV).

Biomechanical parameters (frequency, logarithmic decrement, stiffness) in AII showed individual and mostly positive changes as described in previous article (Tamm et al., 2018). For example, the frequency of *m. sartorius* (Figure 4) and *m. tensor fascia latae* (Figure 6) increased (*p* < 0.05) with wearing GESPA. In AIII, the changes occurred promoted the worsening of GV position. The level of the pressure of elastic traps probably played an important role influencing the muscles’ biomechanical parameters. For example, subject I had low level of the pressure (approximately 550 mN) and the decrease of frequency of *m. tensor fascia latae* during the GESPA wearing period was low.

### 3.2. Feedback to the GESPA and “GVcorrect”

According to the parents, the children wore GESPA on average 6 h a day. Most of the children were eager to wear GESPA at the beginning of the study; but the longer the time, the less the children wished to wear GESPA voluntarily. The children claimed that the GESPA was uncomfortable for the following reasons: the GESPA sank and the need to adjust them tightly (*n* = 4); the discomfort above the knee joint (*n* = 4); the fastening on the midfoot bothered when put on the shoes (*n* = 4); wearing GESPA caused feeling of warmth (*n* = 4); garments did not fit in overweight subject due to their apple type body shape (*n* = 1). However, one third of parents (*n* = 2) claimed that the child was eager to wear GESPA and nothing bothered them. Positively minded parents added that wearing GESPA has brought their child’s lower limbs closer to normal alignment.

Based on the feedback, more comfortable and user-friendly GESPA were created in cooperation with the seamstresses, which the children initially think are comfortable, do not sink, they can easily be worn with winter shoes and are also attractive for children.

According to the results of the questionnaire, the parents agreed that “GVcorrect” was fulfilling its purpose and is convenient to use. Nevertheless, some limitations emerged: the sensors did not stay under the straps (*n* = 6); the sensor charging socket broke down and needed repair by a technician (*n* = 4); the communication between the app and the sensors broke up and the program had to be restarted to restore it (*n* = 6); the degree of pressure did not stay within the recommended range (6). In addition, the parents did not like the fact that the child was forced to carry a smartphone with them. Because the researchers did not have backup sensors (if they were repaired), the child continued to wear GESPA, but the data was not stored in the cloud server.

Researchers evaluated the user-friendliness of monitoring the data in the cloud server (Figure 6). It is clear from the figure whether the stimulus signal remained within the predetermined range during the observation period. According to the survey, not all data reached the cloud server. The amount of data was so large that the server could not receive it. It was possible to monitor the results in real time from the cloud server, but it was difficult to obtain data for data analysis.

Attention was drawn when the child went to another room, the recording was interrupted. To eliminate this, a change was made in the sensor and mobile application programs, which allows data to be stored in the sensor processor memory for up to eight hours. Children are not forced to carry the smartphones with them anymore. The program in the sensor continuously records the pressure sensor signal at one-minute intervals. To save the data, mobile application should place near the sensor (up to 1.5 m), press the save button and then the data will be loaded into the mobile application. Otherwise, the sensor program deletes the first recordings from its memory.

The charging socket for the sensor battery was weakly attached to the first sensors and new sensor housing was made to repair it. Due to this, some children have pauses of up to two weeks in the cloud server recordings, but the children were instructed to wear their GESPA without sensors.

## 4. Discussion

This phase of the project the operation of the equipment in the appropriate environment was checked. The aim was not the number of subjects or corrective parameters, but we focused on the tripartite feedback related to the comfortability of GESPA and reliability and user-friendliness of the “GVcorrect” app. Based on the results, it can be said that GESPA and “GVcorrect” are fulfilling their purpose, but there are still several gaps that need to be improved and developed. Based on the feedback from children and parents, new, more comfortable and user-friendly garments (GESPA) were designed and several updates were made to “GVcorrect” app by the end of the study phase. The new GESPA will be in place in the next phase of research. According to investigators assessment, the use of a cloud server allows control of the adjustment process.

The effectiveness of the alignment depends on the pressure force towards the frontal axis of the knee joint generated by the elastic straps remaining within the selected values. The analyzed data showed that the pressure of the elastic straps of the garments lower than 1000 mN does not cause the correction of the biomechanical axis of the lower limb. However, a compressive force greater than 1500 mN may, according to preliminary estimates, cause discomfort to the child. Thus, the optimal reference range is probably 1000 to 1500 mN and it will be tested in the next phase of the study.

In our view, the determination of IMD to assess a change in the position of a *genu valgum* is complicated because its measurement is related to the child’s standing position (can vary greatly from time to time) and thus, the measurement error is large. The authors of the research do not recommend the use of IMD especially in individual analysis or in a small study group to monitor changes over time. However, the use of IMD is justified for the initial assessment of lower limb alignment.

In children with normal development TFA reaches 168 degrees and stabilizes at 174–175 degrees by the age of six to seven [27]; thus, the alignment of the lower limb should be gently directed with non-invasive method at this particular period. Hopefully, this would help prevent the worsening of *genu valgum* and direct the lower limb alignment in the right direction, which in turn would avoid the need for surgery. At the moment, this is only an assessment by researchers, which must be checked on a larger study group in the next phase of the study. The trend of changing the tibiofemoral angle is informative in assessing the success of the correction process, but it is suggested, that skeletal maturation is the most significant predictive factor in the severity of GV [10], so bone age assessments should be use in next study period.

The tone of *m. sartorius* and *m. tensor fascia latae* increases with the desired level of straps’ pressure, but in general, changes in muscle biomechanical parameters are individual and require also further investigation by a larger study group in the next phase of study.

The next phase of the study project also must: develop comfortable and attractive user-friendly garments (GESPA); to demonstrate the feasibility of the technology in an appropriate simulated environment and to start demonstrating the technology in a working environment. Technical documentation, user, training and maintenance documentation must be prepared. The last task is to apply for a medical device certificate for the device.

There are several limitations in our study. Firstly, we cannot make any final conclusions because of quite small number of participants in our study. However, due to the COVID situation (spring 2020), it would not have been possible to work fully with more participants and their parents. Currently, the “GVcorrect” app only works with android phones, but due to the large number of Apple phones, it would definitely be important to find a way to use the app for users of Apple devices as well.

## 5. Conclusions

The goal of this technology readiness level (simulation should be run in environments that are as close to realistic as possible) was achieved. However, a number of shortcomings need to be addressed before the product can be marketed. Study continues with product development until a medical device certificate is obtained.

## 6. Patents

Vain, A.; Linkberg, R.; Vaher, I.; Tamm, A.-L. Mechanotherapeutic Device and Measurement Method. European patent EP3113729, 2020.

## Figures and Tables

**Table 1:** Anthropometrical and goniometrical data.

Subject	I	II	III	IV	V	VI
Age (y)	5	5	5	7	5	7
Height(cm)	127.9	114.5	122.3	126.3	118.8	129.8
Body mass (kg)	42.2	20.65	23.1	31.7	20.6	30.3
BMI (kg/m^2^)	25.7	15.8	15.4	19.9	14.6	18
GESPA wearing days (n)	93	5	28	42	22	39
IMD AI (cm)	11	9.5	9.5	8	5	5.5
IMD Δ AI–AII (cm)	0.5	−1.5	−1.5	−0.6	0	−0.8
IMD Δ AII–AIII (cm)	−1	−1	−1	−1.6	−0.4	0.1
Median stimulus left leg (mN)	568	810	871	977	874	1441
Median stimulus right leg (mN)	552	829	712	872	858	1286

BMI—body mass index; IMD—intermalleolar distance; AI—first assessment; AII—second assessment; AIII—third assessment; Δ—differences between assessments.

## Data Availability

Not applicable.
